# A Literature Review on Maillard Reaction Based on Milk Proteins and Carbohydrates in Food and Pharmaceutical Products: Advantages, Disadvantages, and Avoidance Strategies

**DOI:** 10.3390/foods10091998

**Published:** 2021-08-25

**Authors:** Jia Xiang, Fenglin Liu, Bo Wang, Lin Chen, Wenjie Liu, Songwen Tan

**Affiliations:** Xiangya School of Pharmaceutical Sciences, Central South University, Changsha 410013, China; 197211040@csu.edu.cn (J.X.); finaliu@koreatech.ac.kr (F.L.); 197211030@csu.edu.cn (B.W.); xiaoxian.xie@csu.edu.cn (L.C.); wenjie.liu@csu.edu.cn (W.L.)

**Keywords:** milk protein, lactose, Maillard reaction, food, medicine, avoidance approaches

## Abstract

Milk has two main components that have high nutritional value—milk protein (casein and whey protein), and lactose. These components are extensively used in various areas, especially in food, i.e., as sweeteners, stabilizers, functional food ingredients, nutritional fortifiers, etc. Non-enzymatic browning refers to a series of chemical reactions between sugars and proteins that make food more appetizing. Non-enzymatic browning reactions include degradation of ascorbic acid, lipid peroxidation, caramel reaction, and the Maillard reaction (MR). The MR, as one of the four non-enzymatic browning reactions, has been well studied and utilized in food fields. Milk protein and lactose, as two main components of milk, have high chemical activities; they are used as reactants to participate in the MR, generating Maillard reaction products (MRPs). The MR involves a condensation reaction between carbonyl groups of various sugars and amino groups of amino acids/proteins. These MRPs have different applications in various areas, including food flavor, food oxidation resistance, drug carriers, etc. This work presents the positive and negative effects of the MR, based on the two main components of milk, used in food and medicine, as well as avoidance approaches to prevent the occurrence of negative effects.

## 1. Introduction

**Figure 1:**
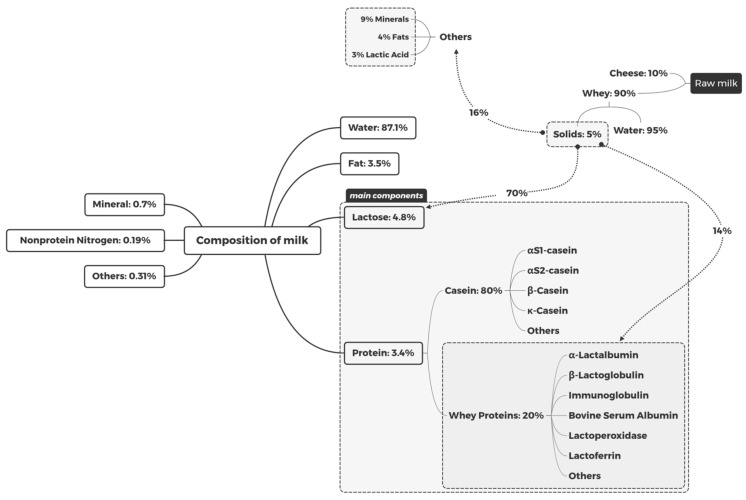
The composition of milk.

Milk, i.e., goat’s milk, pig’s milk, mare milk, cow’s milk, and human milk, involves the secretion of mammary glands from mammals. Currently, cow’s milk is the most popular on the market. In this article, milk refers to cow’s milk. For a long time, humans have associated milk with a high nutritional value. Goulding et al. reviewed the composition of milk [1], as presented in Figure 1. Protein and lactose are the two main components in milk, besides water and fat. Protein consists mainly of 80% casein and 20% whey protein [1]. Consumers also consider the flavor of milk and dairy products, in addition to nutritional value. Cadwallader et al. [2] summarized the flavor and odor of milk and dairy products, which are mainly derived from the related reactions of the components in milk, including lipid oxidation and degradation, glycolysis, protein hydrolysis, heat-induced changes, etc. Heat-induced changes include the occurrence of the MR, in which milk protein and lactose can react to produce a series of MRPs, affecting the quality of milk and dairy products [2]. At present, milk protein and lactose are not only widely used in food fields, but they also play an increasingly important role in non-food fields, particularly in the pharmaceutical industry. The MR plays an important role in the application and development of milk protein and lactose.

**Figure 2:**
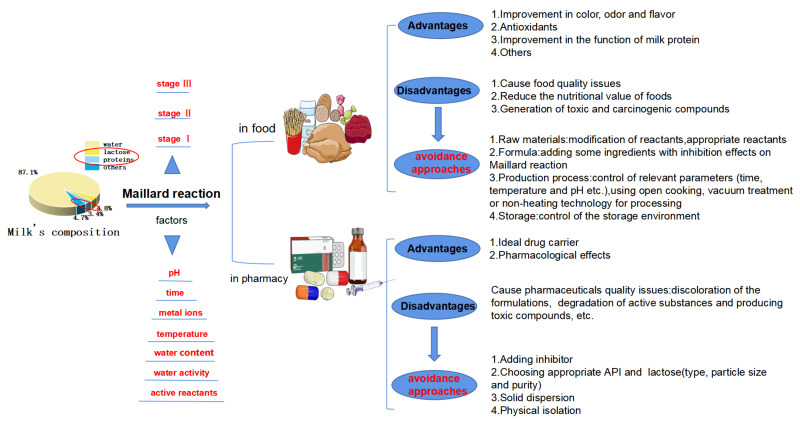
Maillard reaction, based on milk ingredients in food and pharmaceutical products.

The MR was first discovered by Louis Camille Maillard in 1912. It generally refers to nonenzymatic browning, involving the reaction between reducing sugars and proteins [3]. The MR is also called glycosylation widely used in food processing industry. Maillard reaction products are common ingredients in food, which could improve the appearance and taste of food. However, the MR actually has many side effects, of which the most obvious is found in food and pharmaceutical products. At present, many studies show that a series of diseases are related to the intake of MRPs through food [4]. In the pharmaceutical industry, the MR will lead to issues in the preparation stage and in the quality of the products, such as color changes, drug bioavailability, active substance degradation, toxic compounds, etc. [5]. The MR is closely related to the safety and quality of food and drugs. Abd El-Salam et al. [6] investigated the MR between casein and polysaccharides, and El-Shibiny et al. [7] reviewed the effects of glycation on whey protein (which mainly involved the food field). Kumar and Banker [5] summarized the results of various studies involving the MR and pharmaceutical products, which have amine functionality when mixed with carbonyl containing pharmaceutical adjuvants (reducing sugars), including lactose. It is important to control the MR due to the MR having many side effects in the food and pharmaceutical industries. The objective of this paper (Figure 2) is to summarize the influence of the MR on the development and application of milk protein and lactose in milk, in the food and pharmaceutical industries. We also investigated the effective measures to avoid the MR, which may promote further development of milk protein and lactose in the food and pharmaceutical industries.

## 2. Maillard Reaction

**Figure 3:**
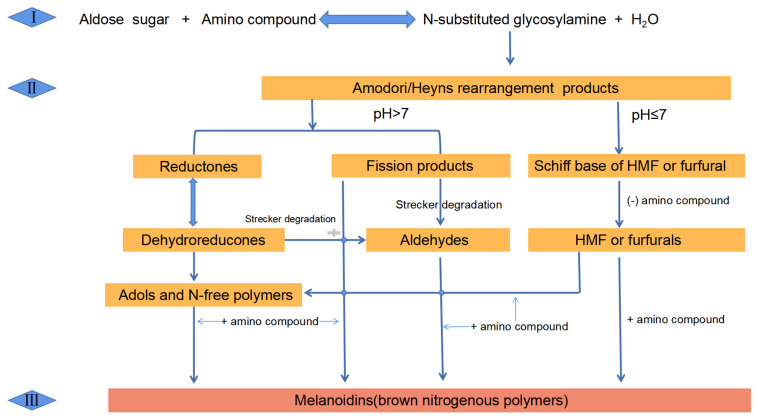
The process of the Maillard reaction.

The MR involves a condensation reaction between a carbonyl functional group (typically aldehyde or ketone group) found in a reducing sugar and α-amino group in amino acids (mainly lysine and/or arginine), amines, peptides, proteins, etc. The nonenzymatic browning reaction is affected by time, water content, water activity, temperature, active reactants, pH, and metal ions in foods [8,9]. Generally, the highest reaction rate is achieved when the water activity is in the range of 0.60–0.85 [10]. The MR directly relates to water content. The reaction occurs easily when moisture content is 30% to 75%; the reaction rate increases with the increase of water content. The rate of MR increased with the increase of pH (3–9) and temperature [11]. In addition, the reaction rate is related to reactants. The order of reactant activity, from high to low, is: reducing monosaccharides > reducing polysaccharides, reducing five-carbon sugars > reducing six-carbon sugars [12]; amine > amino acid > protein [13]. The existence of metal ions, such as iron, copper, and zinc ions, could accelerate the MR [14]. The MR is divided into three stages: initial stage, intermediate stage, and advanced stage (Figure 3) [15]. In the initial stage, firstly, the carbonyl compounds react with the amino compounds to form an unstable Schiff base, which is a reversible process in the MR [16]. Then, the reduction efficiency can be enhanced during the formation of relatively stable Amadori or Heyns rearrangements via double-bond migration and rearrangement processes of the Schiff base [17]. The intermediate stage of the MR involves the degradation of Amadori products, primarily dehydration and deamination of sugar, Strecker degradation, sugar fragmentation, etc. All of these processes are related to pH value. At pH > 7, the reduced ketone products will mainly form by the 2,3-enolization reaction. Whereas, Amadori products mainly produce furfural or hydroxymethylfurfural (HMF) via the 1,2-enolation reaction pathway at pH ≤ 7. In the advanced stage, low molecular weight intermediates undergo a series of reactions, including cyclization, dehydration, rearrangement, post-acetal reaction, isomerization, and other reactions to produce high molecular weight polymers with colored compounds, e.g., melanoidins [15], which are the main colored compounds in coffee, malt, bread, cocoa, and other baked foods. The unique flavors and colors in food are mainly determined by the intermediate and advanced stages of the MR [10]. In the initial stage, the formation of rearrangement products is the vital step in all processes; thus, preventing the formation of rearrangement products could stop subsequent reactions.

## 3. The Maillard Reaction in Food

The MR was first discovered by Louis Camille Maillard. The importance of the MR in food was recognized by Lintner; he believed that the aroma was produced by leucine in malt. The MR in food then received more attention from researchers [18]. The MR is one of four non-enzymatic browning reactions in food (degradation of ascorbic acid, lipid peroxidation, and caramel reaction), an important chemical reaction during food production, processing, and storage. During food production and processing, MRPs can improve the quality of food, while some unfavorable reaction products in food could also be produced by the MR. Milk protein and lactose are currently used to develop MRPs in different food products, which will be introduced in detail in the following section. Table 1 summarizes the MR of milk protein and applications.

### 3.1. Advantages

#### 3.1.1. Improvement in Color, Odor, and Flavor

Lactose is widely used in the food industry due to its physical and chemical properties. Lactose can react with protein and amino acids of foods and produce attractive appearance and taste to various foods, such as dairy products, candy, baking products, sauces, instant drinks, beer, and others [55]. Aromas in certain dairy products that are heat-treated, such as ultra-heat treated (UTH) milk, milk powders, cheese, etc., are mainly produced by the reaction of lactose and milk protein. Studies have shown that these aromas mainly come from MRPs, such as hydroxymethylfurfural, maltol, furfuryl alcohol, furfural, 2-acetylfuran, etc. [56]. In milk powder, the aroma is attributed to the products of the MR, such as maltol, furaneol, and aldehydes [57]. In addition, the MR could improve the taste of food. The MRPs produced by the mixture of amino acids and sugars could not only reduce the stickiness of Cantonese sausages, but also increase the chewiness of Cantonese sausages, thereby helping to improve the flavor of Cantonese sausages [58].

#### 3.1.2. Antioxidants

Studies have shown that MRPs can improve the antioxidant properties of foods, especially for lipid-containing foods that are easily oxidized. The addition of MRPs to food (compared to synthetic antioxidants, such as tert-butyl hydroquinone (TBHQ), butylated hydroxyanisole (BHA), butylated hydroxytoluene (BHT), etc.), is more acceptable for consumers, owing to the toxic effects of synthetic antioxidants on human health [59]. The antioxidant effects of MRPs have various mechanisms, including chelating metal ions, destroying free radical chains and hydrogen peroxide, and scavenging active oxygen. The antioxidant activity is mainly related to intermediate stage of the MR and reaction products at an advanced stage [60]. Yak casein, compared with traditional fresh milk, is more prone to MR under the same conditions. Studies have shown that the MRPs produced by the reaction between Yak/Holstein casein and glucose have antioxidant activities, which could be used as lipid oxidation inhibitors. The MRPs produced by Yak/Holstein casein have improved performance in reducing ability, scavenging ability of DPPH free radical, and chelating ability with Fe^2+^ [31]. MRPs have different antioxidant activities based on the molecular weight differences. A study showed that high molecular weight MRPs have greater metal chelating potential than low molecular weight MRPs, which may be attributed to hydroxyl or pyrrole groups derived from MRPs [61]. A study was conducted on the antioxidant activities of high molecular weight MRPs (melanoproteins) of casein and glucose, and trypsin and peptic hydrolysate of MRPs. The result showed melanoprotein non-hydrolyzed melanoproteins have the highest reducing power, and trypsin hydrolysate products have poor radical-scavenging activity compared to peptic hydrolysates, which is due to the different hydrophilicities of hydrolysates [32]. In the MR system of whey protein-reducing sugar, the antioxidant activities of water-soluble MRPs was studied at different pH values (3–9). The results showed that the properties of browning, reducing ability and scavenging activity of DPPH free radical increased with the increase in pH value [11]. Lactose and whey protein (compared with glucose) have low MR rates, generating more anti-oxidizing material [51]. In addition, the products produced by the MR of lactose (such as baked goods) have antioxidant activities [62].

#### 3.1.3. Improvement in the Function of Milk Protein

Protein is widely used in food due to its properties, such as emulsification, gelation, foaming, and solubility. However, the instability of protein at the isoelectric point, high temperature, and high salt concentration, limits the extensive application of protein. For example, whey protein isolate added in acidic beverages is prone to aggregation, affecting the quality of food. Studies have shown that the derivatization of proteins via the MR could further improve the functional properties of proteins, increasing their value in the food industry [23].

Wang, Qian et al. controlled the reaction conditions to carry out the MR of whey protein and dextran and achieve protein glycosylation and separated MRPs; they compared the different functional properties of whey protein and glycosylated whey protein, such as solubility and thermal stability. The results showed that, when the protein concentration was greater than 4.2%, glycosylated whey protein had higher solubility and thermal stability in a wide pH range than whey protein. The authors related the differences of functionalities to the physical and chemical structural changes of protein (such as resistance to denaturation, a turn to a more acidic isoelectric point reduced sulfhydryl exposure, reduced surface hydrophobicity, and generation of glycosylation sites), leading to reduced intermolecular interactions [63]. The solubility and emulsification of casein could improve by preparing casein–carrageenan (1:2) conjugate by an ultrasonic Maillard dry treatment at 60 °C for 24 h [19]. The conjugate prepared by the MR between whey protein isolate and κ-carrageenan (1:1) at 60 °C for 24 h shows optimal performance as an emulsifier and stabilizer. This natural emulsifier is more popular with consumers compared to synthetic emulsifiers [46]. Gelation is one of the most important functional properties of whey protein. There are two methods to produce whey protein gels—heating and condensation. Spotti prepared heat-induced gels of conjugate through the MR, between whey protein isolate and dextran, and showed improved mechanical properties [43]. Cold-set gels formed from MRPs of whey protein isolate and maltodextrin with a ratio of 1:1 showed enhanced strength of hydrogen bonds, improved gel firmness, water-holding capacity, and reduced gel swelling [44]. The Maillard reaction could also improve the foaming ability of milk protein. Sodium caseinate and maltodextrin of different mass ratios are conjugated through the Maillard reaction, achieving improved solubility of the conjugate, which could mainly be attributed to the marked increase in the thermodynamic affinity for the aqueous medium compared to sodium caseinate, and increased foaming ability and foaming stability, which were mainly attributed to both the increase (of the number density) of the surface active particles and the increase in their relative hydrophobicity, due to the partial destruction of the sodium caseinate associates, as a result of the MR of the maltodextrins and sodium caseinate [64].

The MR could promote the development of milk protein in the food industry by improving the solubility, stability, emulsification, foaming, and gel properties of milk protein. The methods for modifying milk protein through MR mainly include dry heating, wet heat treatment, sonication, pulsed-electric fields, electrospinning, irradiation, high pressure techniques, extrusion, etc. [65].

#### 3.1.4. Others

Κ-casein is the only glycosylated casein in milk. Studies have shown that the coagulation could improve by controlling the degree of glycosylation of milk [66]. Protein could be used as a biodegradable packaging material because of its film-forming property, and is more suitable for food packaging than non-degradable synthetic materials [42,67]. Proteins modified by the MR could produce membranes with better performance in hydrophilicity, plasticity. Cardoso carried out the MR of maltodextrin and casein under certain conditions and prepared film of a casein–maltodextrin conjugate, having improved hydrophilicity, plasticity, and corrosion sensitivity [40]. The MRPs of casein–maltodextrin could be used as nanoencapsulation agents in clear beverages [22].

The antigenicity of protein limits its application and development. Related studies have shown that the antigenicity of milk protein could be reduced by the MR. Taheri-Kafrani et al. studied the recognition ability of MRPs of β-lactoglobulin, and reducing sugar to IgE, in patients with milk allergies. The results showed that the antigenicity of β-lactoglobulin was reduced by the MR, and the degree of reduction was proportional to the degree of the MR [68]. Bu. G et al. studied the effects of MR on the antigenicity of bovine α-lactalbumin at different weight ratios, temperatures, and times via a response surface methodology. The combination of α-LA and glucose through the MR could obviously reduce the antigenicity of α-LA, where the weight ratio of reactants has the greatest influence on antigenicity and the reaction time has the least influence [69].

### 3.2. Disadvantages

Although the MR has brought various unique flavors and other beneficial aspects to food, studies have found that MRPs have a negative impact on foods and the human body.

#### 3.2.1. Cause Food Quality Issues

The MR is one important cause of food spoilage. A moderate MR can bring delicate flavors to food, but the MR is too obvious to bring bitter and scorched tastes to food. The MRPs have different performances in different foods. For example, Strecker aldehydes are very important in forming the flavor in bread, cocoa, and dark beers [70], bringing a peculiar smell to ultra-high-temperature processed milk [71]. The MR between lactose and milk protein is the main cause of quality problems for dairy products. During the milk powder production or storage process, a change of color and the smell of milk powder is induced by the MR when the temperature and moisture are not properly controlled. The main compounds that induce odor are 2-furaldehyde, 2-furfuryl butyrate, alkylpyrazines, and N-ethyl-2-formylpyrrole [57]. Studies have shown that spray-dried WPI-ascorbic acid will undergo MR to produce a red substance of formyl threosyl pyrroles during the processing and storage stages, which affect the quality; these red pigments are also very unsafe, especially for infants and babies [72]. Fat oxidation, caramelization, and the Maillard reaction are the main reasons for the change of volatile components in processed cheese during heat treatments, producing volatile off-odor components in cheese. Among them, odorous compounds from the Maillard reaction, including maltol and furaneol, mainly contribute to the off-odor cheese. When the heating temperature of cheeseexceeded 120 °C for 5 min, it will produce peculiar smell, which was mainly produced by Furaneol and maltol, which were formed by the degradation of lactulose lysine during MR [73,74]. The formation of Nε-carboxymethyllysine (CML) and Nε-carboxyethyllysine (CEL) produced via the advanced Maillard products is the main cause of color changes in cheese products. As the content of CML and CEL increases, the color of cheese gradually changes from white to yellow [75]. Many proteins in food undergo protein cross-linking during processing and storage, resulting in the change of protein properties, affecting the quality of foods. Usually, protein cross-linking is induced by the interaction between protein and protein. In addition, Maillard products could react with protein, leading to protein cross-linking. The protein cross-linking in dairy products occurs through the formation of MRPs or dehydroalanine, causing discoloration of dairy products and leading to various issues, such as poor performance in solubility, foaming, and emulsification. MRPs are the main causes of protein cross-linking in the presence of lactose [76,77]. Pellegrino et al. studied the mechanism of β-casein cross-linking and aggregation in the presence and absence of glucose, and found that β-casein aggregation only occurs in the presence of glucose [78].

#### 3.2.2. Reduce the Nutritional Value of Foods

The MR between lactose and lysine in food will reduce the digestibility and bioavailability of lysine [79]. Studies have shown that the occurrence of the MR in milk powder results in the loss of vitamins and milk proteins in the milk powder. The degree of damage is related to the progress of the MR and is mainly affected by storage time, temperature, and humidity. Furthermore, the browning reaction of milk powder in N_2_ is faster than that in O_2_ [80]. Compared with normal milk powders, infant formula milk powder has a higher degree of damage [81]. Flavored milk products generally contain added sweeteners, in addition to lactose. With long-term storage of products after ultra-high temperature heating treatment—the milk protein will experience a loss of amino acids, reducing the nutritional value owing to the occurrence of the MR. Geicu et al. used proteomic and immunochemical approaches to study the changes of the MR of milk protein in flavored drinks. Two-dimensional electrophoresis (2-DE) profiles and western blots of glycated total casein show that αs2-casein and β-casein are mainly involved in the generation of advanced glycation end-products through the MR [82]. In addition, MRPs will also affect the bioavailability of trace elements, such as reducing the bioavailability of iron, phosphorus, and magnesium [83,84,85].

#### 3.2.3. Generation of Toxic and Carcinogenic Compounds

During food processing and storage, the MR could produce toxic substances, such as acrylamide, heterocyclic amines, furan, 5-hydroxymethylfurfural, and carboxymethyllsine (CML) [86,87]. These compounds have neurotoxicity, genetic toxicity, carcinogenicity, reproductive toxicity, liver toxicity, and immunotoxicity, which seriously affect human health [4]. Acrylamide and furan have notably been classified by the International Agency for research on Cancer as “probably” carcinogenic to humans. The MRPs produced by casein and reducing sugars are carcinogenic. The carcinogenicity of MRPs generated by different reducing sugars is different. Among them, reducing sugar containing glucose and galactose has higher carcinogenicity, while reducing sugar involving lactose and galactose has lower carcinogenicity. These differences are mainly attributed to the differences of reaction mechanisms [88]. The MR of WPI-glucose promote prostate cancer proliferation, while early glycation products promote the prostate tumor proliferation indirectly through modulating macrophages, while advanced glycation end-products (AGEs) have a direct effect [89]. Studies have shown that the MR also occurs in vivo to produce AGEs. This reaction belongs to the normal metabolism of the human body; however, it may cause a series of diseases when the level of AGEs in our body tissues is too high. MRPs are related to various inflammations, and may cause renal failure, diabetes [90,91,92], atherosclerosis [93], chronic heart failure [94], Alzheimer’s disease, Parkinson’s disease, and other diseases [95]. These diseases occur mainly by binding to cell surface receptors or crosslinking with proteins in vivo, changing their structures and functioning, and promoting the development of oxidative stress and inflammation [91]. In addition, exogenous AGEs from diet intake may accumulate in the body and affect human health [96]. The CML content in infant formula milk powder is 70 times than that of breast milk. Study results showed that CML content in the plasma of infant formula milk powder is 46% higher than that in breast milk [97]. Thus, it is essential to control the formation of unnecessary MRPs in food.

## 4. Maillard Reaction in Drugs

In the pharmaceutical industry, the MR causes problems in pharmacy formulations, affecting the safety and effectiveness of drugs. However, the MR could bring some positive effects to pharmacy formulations.

### 4.1. Advantages

#### 4.1.1. Ideal Drug Carrier

Milk protein is an ideal carrier for biologically active substances, with good functionality, biocompatibility, and biodegradability [98]. For example, a stabilized nanoemulsion of whey protein could be used as a delivery system for insoluble curcumin [99]. Casein could self-assemble into stable micelles, be used as carrier for poorly stable or hydrophobic drugs, such as vitamin D2 [100], naringenin (the main hydrophobic flavanone in grapefruit) [101], and β-carotene [102]. However, the physical and chemical properties of milk protein limit its practical application as a carrier material. At present, studies found that milk protein could form a more stable glycosylated protein through the MR, fully exerting its functions as a drug carrier.

Coenzyme Q10 is one of the substances involved in the electron transport chain and aerobic respiration in the mitochondria. Many diseases are related to the insufficiency or lack of coenzyme Q10; thus, coenzyme Q10 supplementing is necessary. This drug has poor water solubility, and with the general dosage received from the pharmacy, it is hard to achieve the desired effects, in terms of solubility, dissolution in vitro, and bioavailability. It could be encapsulated in drug delivery systems to improve these properties. Studies have found that dextran and casein could be covalently bonded through MR to prepare stable casein glycosylated micelles as the carrier of coenzyme Q10, which could significantly improve the solubility, dissolution in vitro, and bioavailability [30]. The efficiencies as a carrier for curcumin, with poor water solubility of casein, and the Amadori rearrangement product of the casein–dextran through the MR, are compared, and results show that the combination of curcumin and MRP has higher affinity and better performance in improving the stability of curcumin and scavenging free radical activity than that of casein micelles [28]. The MRPs obtained via fermentation of whey protein and isomaltooligosaccharide (4:1) at 90 °C for 4 h have a higher encapsulation rate, better protection for *lactobacillus rhamnosus*, and are more suitable for enveloping usage [47].

Anthocyanins with poor stability are sensitive to heat and acid. The MRP produced by whey protein isolate and glucose could improve the thermal stability and antioxidant properties of anthocyanins [48].

#### 4.1.2. Pharmacological Effects

Studies have shown that melanin, a product of MR, has antioxidation, antibacterial, probiotic, and antitumor pharmacological activities in the human intestine, which is beneficial to human health [103,104]. Kang et al. have found that the intake of fermented Maillard-reactive whey protein could enhance the function of the natural killer (NK) cell and human immunity [105]. Through quantitative real-time PCR (qRT-PCR) and in situ hybridization analysis, researchers found that the products produced by the MR of casein and the MRP generated via fermented by lactobacillus rhamnosus 4B15 have pharmacological effects in preventing testicular dysfunction caused by chronic stress [106]. Glucose-whey protein concentrate (WPC) conjugates compared to WPC demonstrated immunomodulatory effects and increased phagocytic activity of RAW264.7 cells [107]. The MR between the excipient lactose and the drug does not guarantee the safety and efficacy of drugs. Therefore, the MR between the excipient lactose and the drug substance is not expected. However, the product of the MR may have pharmacological effects and is worthy of being developed. Pregabalin works in the central nervous system, and the primary amine is present in pregabalin. One of the excipients in the original formulation is lactose. During its production and storage, we focused on avoiding the MR between pregabalin and lactose to ensure the quality of the formulation. However, recent studies found that the MRP of pregabalin and lactose have similar pharmacological activities to pregabalin [104].

### 4.2. Disadvantages

The excipient lactose has been widely applied in pharmaceutical preparations owing to a wide range of sources, low costs, safety, mild taste, low sweetness, hygroscopicity, etc. [108,109]. However, the MR between lactose and amino-containing drugs has always been an obstacle restricting the extensive application of lactose and its pharmaceutical formulations. At present, many pharmaceutical companies choose to replace the lactose of the original formulation with another excipient that does not react with the active pharmaceutical ingredient (API) when making generic drugs. This will affect the efficacy of the drug, and increase the difficulty of the evaluation consistency of drugs. The MR between lactose and amino-containing APIs will not only cause discoloration of the formulations, but also lead to degradation of active substances, producing toxic compounds, etc. There are seven degradation products observed in formulated pregabalin, which affect the quality of pregabalin. Michael J. Lovdahl et al. studied the synthesis, isolation, and spectral characterization of the degradation products of formulated pregabalin in detail [110]. As a primary amine drug, aminophylline will react with lactose via MR when mixed with lactose at a ratio of 1:5 (*w*/*w*) and stored at 60 °C. After three weeks, brown mixture is formed [111]. Research works have found that the degradation of amlodipine besylate is mainly caused by the MR between lactose and the APIs [112]. In animal experimental tests, it was found that the MR between lactose and nebivolol will induce the loss of pharmacological activity of nebivolol [113]. In addition to reaction of lactose with drugs, the HMF remaining in lactose will further undergo MR in formulations, affecting the stability of drugs. Compared with other lactose products, spray-drying lactose will produce a large amount of 5-(hydroxymethyl)-2-furaldehyde (HMF) during its production [114]. Factors affecting MR include temperature, moisture of pharmaceutical formulations, as well as the content of lactose. When the content of lactose exceeds 95%, the possibility of MR greatly increases [115]. Studies have shown that the excipient lactose could react with gabapentin/baclofen freeze-dried formulations [116], baclofen tablets and granules [117], fluoxetine HCl [118], doxepin [119], acyclovir [120], and sertraline [121]. The incompatibility of the raw material drugs causes the MR, leading to quality problems. Therefore, it is necessary to strictly control the MR between the excipient lactose and the drug to ensure the stability of drugs.

## 5. Maillard Reaction Avoidance Approaches

Some MRs have adverse effects on foods, pharmaceutical formulations, and human health. Thus, it is of great importance to reduce the occurrence of MRs with side effects. For the food industry, avoiding the generation of toxic compounds is the key goal during food production, processing, and storage. In the pharmaceutical industry, it is the main task to avoid the MR between the excipient lactose and the APIs, as well as to control the generation of impurities.

At present, there are many methods to effectively avoid the MR, mainly according to the mechanisms and characteristics. Effective avoidance approaches could be achieved and determined for food from the following four aspects: raw materials, formula, production process, and storage. Modifying raw materials to reduce their reactivity could inhibit MR.Adding starter to cheese to ferment reducing sugar into non-reducing sugar can prevent browning [122]. The modification of amines could also inhibit MR, i.e., modification of amines on lysine residues in a whey protein isolate (WPI) by acetylation has been shown to protect lysine from further modification during storage at 50 °C [123]. Choosing appropriate reactants and reaction conditions could reduce the generation of toxic substances produced by the MR. Ho-Young Park et al. studied the reaction of casein and reducing sugars (glucose, tagatose, and xylose) in milk at different temperature (75 °C, 120 °C) and determined the optimal sweetener of dairy products by the production of MRPs (Ne-(-carboxymethyl)lysine). Experimental results illustrated that the CML concentration produced by tagatose at 75 °C is lower than that produced by glucose and xylose. There is no significant difference among three reducing sugars at 120 °C. Therefore, tagatose is more suitable as a sweetener added to dairy products [124]. Calcium ions and epicatechin molecules could inhibit the MR to minimize heat damage between casein (aqueous solution or dry state) and glucose through hydrophobic and ionic interactions. Meanwhile, it still has inhibiting effects of the MR, even in a wide temperature range, from 70 °C to 150 °C [125]. In addition, there are many other approaches to avoid the occurrence of MR, including: (1) adding some ingredients with inhibition effects on the MR, such as acidic ingredients, lemon juice, phenolic compound, vitamin, cations (salts), aminoguanidine, and enzyme (fructosamine oxidase, fructosamine kinase, and hexose oxidase), etc. Attention should be paid to the amount of inhibitor added. Some additives with too-high concentrations will produce peculiar smells. (2) Strictly controlling the relative parameters, such as time, temperature, pH, etc., during the production process. (3) Using open cooking, vacuum treatment, non-heating techniques, strict control of food storage environment, etc. [126,127,128,129].

In 1967, the inorganic bisulfite was first proposed and used to inhibit the MR between drugs and lactose by Griffin and Banker [130]. Hammouda and Salakawy et al. studied the effect of sodium bisulfite concentration on the browning rate of neomycin-lactose tablets. The results showed that adding appropriate concentrations of sodium bisulfite could effectively inhibit the MR between lactose and the drugs [5]. With the development of the pharmaceutical industry, available measures used to avoid the occurrence of the MR have become increasingly diverse and effective. A solid dispersion is used to avoid the MR by making pharmaceutical preparations into intermediate solid forms. During the production process of the non-steroidal anti-inflammatory drug, the ketorolac tromethamine tablet, the raw material was made into a solid dispersion and then mixed with lactose, achieving compatibility of mixing tablets of lactose and amino-containing APIs [131]. Showa proposed that the MR could be reduced by reducing the physical contact area via coating soluble high-molecular substances (dextrin, gelatin, sodium carboxymethyl cellulose, etc.) to APIs containing amino groups [132]. Currently, the main approach to reduce MR is reducing the physical contact area of the original formula based on the key idea of the coating principle. For example, the preparation process of the entecavir tablet used in the treatment of chronic adult hepatitis B involves mixing the APIs with a solid dispersion carrier to granulate, and then adding the lactose and other excipients into mixtures to make tablets. This process greatly reduces the physical contact area between the APIs and lactose, improving the dissolution of drugs, and reducing the impurities of formulations compared with the wet granulation process [133]. For compound tablets, it is an effective method to minimize the occurrence of the MR, to mix the tablets composed of the mixture of excipient lactose and APIs, with poor or no reactivity, and drugs with high reactivity for tableting. In addition to different preparation methods, some process parameters, such as temperature, pH, humidity, different specifications of APIs, and lactose, will affect the MR between lactose and drugs. Thus, the occurrence of the MR could be avoided by controlling these factors. In the preparation of the antibacterial drugs (cephradine capsule), the method of refining APIs is adopted to inhibit the MR. By comparing the preparation of unrefined APIs, experimental results indicated that the impurity content of refined APIs significantly reduced and successfully achieved the inhibition effects on MR [134]. During the study of drugs for the nervous system (pregabalin capsule), researchers have found that the particle sizes of the lactose impact the MR, and the most suitable particle size for lactose in this study was the D50 particle size (180 to 200 um) [135].

## 6. Conclusions and Prospect

The Maillard reaction (MR) of major components in milk is widespread in the food and pharmaceutical industries. MRPs induced by milk protein and lactose in food could bring attractive colors, fragrances, and improve the taste of food. Furthermore, Maillard reaction products (MRPs) with antioxidant properties have more advantages than synthetic antioxidants used as natural antioxidants in food. Milk protein has been widely used in food due to its unique properties, while its application was limited by its high sensitivity to its surroundings. The MR could improve the functionality of milk protein and expand its usage in food. However, the MR also has negative effects on food, causing color and odor changing, reducing the nutritional value of food, and producing toxic substances, which are harmful to human health. For pharmaceutical formulations, milk protein could be used as a drug carrier through the MR. In addition, the products of MR in vivo have various pharmacological activities, including antioxidative properties, antibacterial activities, antitumor properties, improved immunity, and preventive effects on chronic stress, such as testicular dysfunction caused by chronic stress. However, most of the MR induced by the excipient lactose causes quality issues (in regard to drugs), which has caused obstacles in the pharmaceutical industry. It is essential to inhibit the occurrence of the MR with adverse effects on the food and pharmaceutical industries. Effective avoidance approaches could be achieved and determined for food from the following four aspects: raw materials, formula, production process, and storage process. The objective is to control the production of toxic compounds while ensuring the food tastes good. The main methods used to avoid the MR between the excipient lactose and the amino-containing APIs include adding inhibitors, preparation of a solid intermediate dispersion, reducing the physical contact area between the excipient lactose and drugs, selecting appropriate specifications of APIs, and controlling the process parameters. In the future, on the one hand, we are supposed to develop the applications of the MR of milk protein and lactose, which are beneficial to us. On the other hand, there are now many methods to control the MR in food, with fewer methods to avoid unfavorable MRs in drugs, so it is of great significance to study the circumvention method of the MR in depth, especially in the pharmaceutical industry. Future works should focus on more controllable, economical, and environmentally friendly methods to inhibit the occurrence of the undesirable MR.

## Figures and Tables

**Table 1:** The Maillard reaction of milk protein and applications.

	Reaction		Property	Application	Reference
Casein	Carrageenan	60 °C/24 casein:carrageenan = 1:2	Improving emulsification and thermostability.	An effective carrier of red pigment from paprika.	[19]
	Maltodextrin	60 °C/79%(RH)/120 h casein:maltodextrin = 1:1	Improving solubility and emulsification.	Food emulsifiers or soluble protein additives.	[20]
		60/79%(RH)/72 h/pH 4.8 maltodextrin:sodium caseinate = 0.4; 1; 2; 5	Improving solubility, thermostability, and foaming	Biodegradable packaging materials that increased sensibility for erosion in acid environment	[21]
		90 °C/8 h/pH = 7.8 bovine casein aqueous dispersion 5% (*w*/*v*), maltodextrin 10% (*w*/*v*)	Improving thermal stability, the films with modified casein presented as more plastic, hydrophilic, and sensitive to erosion in acid environment.	Increasing casein films applications.	[22]
		60 °C/79%(RH)/4, 6, 8 h maltodextrin:sodium caseinate = 4:1	Improving emulsification and thermostability.	Nanoencapsulation of hydrophobic nutraceuticals in clear beverages.	[23]
		Spray drying at an inlet temperature of 170 °C, a feed rate of 12%, and an airflow rate of 35 m^3^/h. 80 °C/79%(RH)/2.5 h	Improving the encapsulation performance.	A better emulsifier.	[24]
		Freeze-dried over a saturated KBr solution. 60 °C/79%(RH)/4 days	Improving storage stability and freeze–thaw stability	Specialty functional food ingredients.	[25]
	Dextran	60 °C/79%(RH)/48 h casein:dextran = 1:5/7.5	Improving solubility, thermostability, and emulsification.	Increasing the applicability of casein as a protein ingredient in different food systems.	[26]
		60 °C/78.9%(RH)/pH = 4.6,8 β-casein:dextran = 1:8~8:1	Improving emulsifying ability.	A better emulsifier in acidic solution.	[27]
		−50 °C/ lyophilized/3 days, and then 60 °C/79%(RH)/20 h casein:dextran = 4:1	Higher structural stability.	A protective carrier for bioactive curcumin.	[28]
		60 °C/79%(RH)/8 h	Improving stability and release properties of emulsions.	Emulsifier that encapsulated vitamin B12.	[29]
		60 °C/78.9%(RH)/20–24 h casein, dextran = 5 g, 35 g	Higher structural stability.	Nanostructured delivery system with appropriate stability in acidic food and beverages carrier for coenzyme Q10.	[30]
		60 °C/8 h sodium casein:dextran = 1:2	Improving emulsifying activity and the stability.	Increasing the applicability in food systems.	[21]
	Glucose	100 °C/3 h	Improving antioxidant activity.	Food antioxidant.	[31]
		102 °C/130 min casein:glucose = 1:2			[32]
		55 °C/28 day/pH = 7 casein:glucose = 2:1			[33]
		55 °C/7 days Sodium casein:glucose = 1:5			[34]
	D-fructose	55 °C/28 days/pH = 7 casein:sugar = 2:1			[33]
	D-ribose				
	Lactose	55 °C/7 days Sodium casein:lactose = 1:5			[34]
	Pectins	60 °C/80%(RH)/10 days/pH = 7 casein:pectin = 1:3	Improving thermostability and emulsification.	A better emulsifier.	[35]
		60 °C/79%(RH)/48 h sodium casein:pectin = 1:1			[36]
	Locust bean gum	54~96 °C/1 or 3 h sodium casein:locust = 0.3~1.0			[37]
	Fructose glucose	60 °C/67%(RH)/24 h casein:fructose/glucose = 1:0.2	Improving viscosity.	A valuable food ingredient.	[38]
	Lactose	60 °C/67%(RH)/48 h casein:lactose = 1:0.2			
	Ribose	60 °C/67%(RH)/24 h casein:ribose = 1:0.02			
	Lactose galactose	50, 60 °C/aw= 0.67/pH = 7,	Improving the viscosity and gelling properties.	Food ingredient.	[39]
		60 °C/4 h (lactose) 60 °C/8 h (galactose) Sodium casein:lactose/galactose = 1:0.2	Improving emulsifying activity and the stability.	A better emulsifier.	[40]
Whey	Pectins	60 °C/80%(RH)/10 days/pH = 7 whey:pectin = 1:3	Improving emulsifying activity and emulsion stability.	A better emulsifier.	[35]
	Dextran	60 °C/63%(RH)/2, 5, 9days 12% (*w*/*w*) WPI, 7.2% (*w*/*w*) dextran	Changing the rheological properties increasing time and temperature of gelation.	Food ingredient.	[41]
		50, 55, 60 °C/48, 72, 96, 120, 140 h/aw = 0.49 WPI:dextran = 1:4	Improving solubility and thermostability.	Acidic beverages.	[42]
		60 °C/63%(RH)/2, 5, 9 days 12% (*w*/*w*) WPI, 3.6~10.8%(*w*/*w*) dextran	Improving gel mechanical properties.	As a food ingredient.	[43]
		90 °C/2.5 h mixed solution(WPI:dextran = 1:1)	Improving gel firmness and water-holding capacity reduce the extent of gel swelling.	Food ingredient for obesity treatment.	[44]
		60 °C/aw = 0.44 β-lactoglobulin/α-lactalbumin /bovine serum albumin:dextran = 1:2	Improving emulsifying activity and thermostability.	Broadening their food applications due to their improved heat stability at acidic pH.	[45]
	κ-carrageenan	65 °C/12 h milk protein isolate:κ-carrageenan = 1:1	Improving physical properties of oil-in-water emulsions.	A natural emulsifier and stabilizer in the food industry.	[46]
	Isomaltooligosaccharide	90 °C/4 h whey protein:isomaltooligosaccharide = 4:1	Higher structural stability.	A better protection of Lactobacillus rhamnosus.	[47]
	Glucose	80 °C/79%(RH)/18 h/pH = 6.0 WPI: glucose = 1:1	Higher structural stability.	Improvement in the color stability and antioxidant capacity of the anthocyanin.	[48]
		60 °C/79%(RH)/0~7 days whey protein isolate: glucose = 1:1 dry-heating conditions	Improving thermal stability and antioxidant properties.	Food antioxidant.	[49]
	Lactose/glucose	50 °C/65%(RH)/51, 96 h β-Lactoglobulin: lactose/glucose = 1:10/100	Improving foaming properties.	As a food ingredient.	[50]
		55 °C/7 days whey: lactose/glucose = 1:5	Improving antioxidant activity.	Adding in functional dairy products.	[34]
		37 °C, 60 °C/aw = 0.52			[51]
	Inulin	70 °C/2, 4, 6 h wet-heating method whey protein isolate: inulin = 1:1		As natural antioxidants in food products.	[52]
	D-arabinose,	60 °C/72 h β-Lactoglobulin:sugar = 1:1		As functional ingredients used in formulated food.	[53]
	D-galactose				
	D-glucose				
	D-lactose				
	D-rhamnose				
	D-ribose				
	Xylose	50 °C/7 days/pH = 3~9 whey protein isolate: sugar = 2:1		As natural antioxidants in food products.	[11]
	Glucose				
	Fructose				
	Lactose				
	Maltose				
	Sucrose				
	Chitosan	40 °C/79%(RH)/7days β-Lactoglobulin:chitosan = 1:2	Improving emulsifying properties and antioxidant properties	Food ingredient.	[54]

Abbreviations: RH—relative humidity; pH—potential of hydrogen; aw—water activity; WPI—whey protein isolate; KBr—potassium bromide.

## Data Availability

Not applicable.
